# Functional outcomes after transanal total mesorectal excision (TaTME): a random forest analysis to predict patients’ outcomes

**DOI:** 10.1007/s10151-023-02775-5

**Published:** 2023-03-05

**Authors:** F. Tirelli, L. Lorenzon, A. Biondi, I. Neri, G. Santoro, R. Persiani

**Affiliations:** General Surgery Unit, Fondazione Policlinico Universitario Agostino Gemelli IRCCS, Catholic University, Largo Francesco Vito 1, 00168 Rome, Italy

**Keywords:** Transanal total mesorectal excision, TaTME, Functional outcomes, LARS score, Random forest

## Abstract

**Purpose:**

Anorectal, sexual, and urinary dysfunction are common issues after rectal cancer surgery, although seldom explored. The primary aim of this study was to investigate postoperative anorectal functional results.

**Methods:**

Patients with mid/low-rectal cancer treated with transanal TME (TaTME) with primary anastomosis with/without diverting stoma between 2015 and 2020 were reviewed and selected if they had a minimum follow-up of 6 months (from the primary procedure or stoma reversal). Patients were interviewed using validated questionnaires and the primary outcome was bowel function based on Low Anterior Resection Syndrome (LARS) scores. Statistical analyses were performed to identify clinical/operative variables correlated with worse outcomes. A random forest (RF) algorithm was computed to classify patients at a greater risk of minor/major LARS.

**Results:**

Ninety-seven patients were selected out of 154 TaTME performed. Overall, 88.7% of the patients had a protective stoma and 25.8% reported major LARS at mean follow-up of 19.0 months. Statistical analyses documented that age, operative time, and interval to stoma reversal correlated with LARS outcomes. The RF analysis disclosed worse LARS symptoms in patients with longer operative time (> 295 min) and stoma reversal interval (> 5.6 months). If the interval ranged between 3 and 5.6 months, older patients (> 65 years) reported worse outcomes. Finally, no statistical difference was documented when comparing the rate of minor/major LARS in the first 27 cases versus others.

**Conclusion:**

One-quarter of the patients developed major LARS after TaTME. An algorithm based on clinical/operative variables, such as age, operative time, and time to stoma reversal, was developed to identify categories at risk for LARS symptoms.

**Supplementary Information:**

The online version contains supplementary material available at 10.1007/s10151-023-02775-5.

## Introduction

Since the first reported case in 2010 [1], transanal total mesorectal excision (TaTME) has been enthusiastically welcomed among the surgical community, as an innovative technique for low rectal cancer treatment.

Although the technique has been criticized for the higher rate of local recurrence reported by few authors [2, 3], undoubtedly the core benefit of the transanal approach is an improved visualization of the surgical planes in the mid- and low rectum, which allows a more precise mesorectal dissection in a narrow pelvis, increases the rate of sphincter-saving procedures, and results in reduced conversion rates [2, 4–6].

However, and despite the advantages in terms of surgical dissection and pelvic neural plexa preservation that this technique could offer, several concerns exist regarding the functional outcomes after TaTME [7–10].

Indeed, and independently from the surgical technique, restorative surgery with ultra-low anastomosis [11–13] and a history of neoadjuvant therapy [14, 15] are correlated with the development of postoperative anorectal dysfunction. Literature in this field reports that 50–80% of patients undergoing surgery for rectal cancer have symptoms of bowel dysfunction, such as urgency and/or incontinence, all included in the spectrum of the low anterior resection syndrome (LARS) [5, 16, 17]. Genitourinary problems, such as urinary incontinence, erectile and ejaculation dysfunction, and dyspareunia are also reported, but inadequately explored [5, 18, 19], with TaTME showing similar results in terms of urinary and sexual outcomes to other mini-invasive techniques [20].

On this basis, the primary aim of this study was to investigate the postoperative anorectal functional results in patients treated with TaTME for mid or low rectal cancer in a high-volume institution. The primary objective was to identify the categories of patients at a greater risk of poor anorectal function on the basis of clinical and operative data. In addition, the entire spectrum of bowel, urinary, and sexual functional outcomes was evaluated as a secondary outcome.

## Materials and methods

This research has been designed and reported according to the STROBE criteria for observational studies [21] (Supplementary Table 1). The research protocol has been notified to the local IRB. Patients who underwent TaTME for rectal cancer at our unit between May 2015 and November 2020 were eligible for enrollment. The unit is part of the Surgical Department of Fondazione Policlinico Universitario Gemelli in Rome, a University Research Hospital performing more than 170 rectal cancer resections per year (https://pne.agenas.it/risultati/tipo5/tab_strT5.php?ind=120&tipo=5&area=2). Of note, since 2015 the TaTME technique has been introduced at our unit and it has become the treatment of choice for patients with low and mid rectal cancers (1–6 cm and 7–11 cm from the anorectal junction, respectively).

Patients with primary anastomosis were reviewed, including those with and  without stoma diversion. For the purpose of this analysis, patients with a minimum follow-up of 6 months (from stoma reversal or from the primary procedure) were selected. E  Patients who underwent TaTME without primary anastomosis (Hartmann’s/Miles procedure) and patients who did not undergo stoma reversal were excluded along with those who were unable or unwilling to participate in functional outcome investigation. Clinical [age, sex, body mass index (BMI), Charlson Index, American Society of Anesthesiologists (ASA) score, tumor location, neoadjuvant treatments], pathological (American Joint Committee on Cancer Stage), operative (type and shaping of anastomosis, stomas, operative time) and postoperative data (Clavien–Dindo complications, hospital stay, readmissions), and functional outcomes were collected in a prospectively maintained database.

*Preoperative assessment and TaTME technique* The multidisciplinary management of all patients with rectal cancer treated at the institution is discussed during weekly multidisciplinary team (MDT) meetings. In brief, patients with cT3–cT4a N0 disease, or those staged cTN^+^, are usually scheduled for neoadjuvant chemoradiotherapy, consisting of 4 weeks of radiotherapy (total dose of 56 Gy) plus concomitant 5 fluoro-uracil, followed by delayed surgery after at least a 6-week interval. Short-term radiotherapy (total dose of 25 Gy), followed by immediate or delayed surgery, is usually applied for patients unfit for chemotherapy.

The surgical technique has been standardized since its adoption [22], and the combined transanal/transabdominal procedure (Cecil approach) was introduced after the first eight sequential patients; all cases were performed by the same surgical team.

Although a diverting stoma is performed in the vast majority of the cases, the decision on whether to perform or not is at surgeon’s discretion, based on clinical features (i.e., comorbidities, tumor height, neoadjuvant therapy, possible need for adjuvant therapy) and intraoperative findings (i.e., intraoperative anastomotic integrity tests positive for technical defects).

*Functional outcomes* Functional outcomes were assessed using the following items: the Cleveland Clinic Fecal Incontinence (FI) Score (CCFIS, also known as Wexner scale), the low anterior resection syndrome (LARS) score, the long form module of International Consultation on Incontinence Questionnaire for Male Lower Urinary Tract Symptoms (ICIQ-MLUTS), the long form of International Consultation on Incontinence Questionnaire for Female Lower Urinary Tract Symptoms (ICIQ-FLUTS), the International Index of Erectile Function (IIEF), and the Female Sexual Function Index (FSFI).

At the beginning of the study periods, questionnaire answers were collected by face-to-face interview during postoperative follow-up. Subsequently, because of limitations due to COVID-19 pandemic, telephone interviews were performed. To reduce potential bias, patients were called by same-gender physicians. A detailed explanation of the questionnaires and all the spectrum of symptoms investigated is available in Supplementary Table 2.

*Primary outcome* The primary outcome was bowel function, primarily based on LARS scores. Other functional data including urinary and sexual functions were collected and analyzed as secondary outcomes.

*Statistical analysis* Preliminary descriptive analyses were performed considering the distribution (mean ± standard deviation, or median and interquartile range) and frequencies of the variables (percentages).

Association of the results for the LARS score (categorized as: no LARS, score 0–20; minor LARS, score 21–29; major LARS syndrome, score 30–42), and the Wexner score (categorized as: no FI, score 0; minor FI, score 1–8; average and complete FI, score 9–14 and 15–20, respectively), urinary and sexual functional results were evaluated using Mann–Whitney *U* test, *t*-test and Pearson’s chi-squared test, with Bonferroni correction when required.

A supervised machine learning approach was then computed to test the prediction of variables for favorable (no LARS syndrome) versus unfavorable outcomes (presence of minor/major LARS syndrome). On this basis, the data set was randomly partitioned (80% training set, 20% test set) and an implementation with a 10 k-fold cross-validation method was performed, to include simple decision trees (DT) per each fold. To increase accuracy in the analyses, a random forest (RF) classification model was designed by the aggregation of many decision trees [22]. Finally, the entire model was checked for control over the prediction using the confusion matrix.

Also, to explore the impact of the learning curve, the LARS scores reported in the first cohort of patients treated with TaTME were compared with those obtained in the subsequent cases. The cut-off value for the learning curve was based on the number of patients required to decrease the rate of anastomotic leaks, as previously reported [23]. The comparisons were made using the chi-squared test with Yate’s correction for continuity and the analysis of standardized adjusted residuals (to verify if the differences between observed and expected values depended on random fluctuations). A post hoc analysis was then performed to evaluate the power of this test, setting the size effect at 0.3, with an alpha-error of 0.05.

The analyses were performed using R software (4.1.1), implemented with “CART,” “Tree,” “RandomForest,” and “Rpart” packages, whereas for the post hoc analysis the following packages were used: “chisq.posthoc.test” and “pwr”. All packages were downloaded from the CRAN Mirror Repository (https://cran.r-project.org/mirrors.html). All tests were two-sided with a significance level set at *P* < 0.05.

## Results

**Fig. 1:**
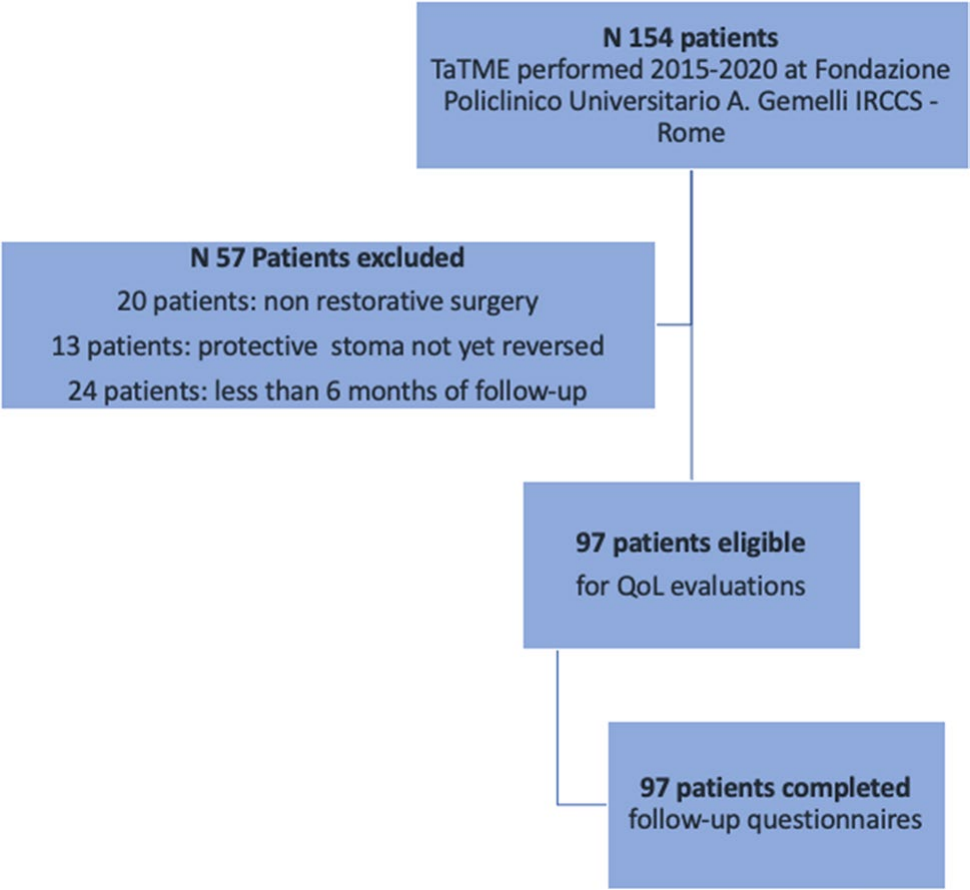
Flow-chart of patients’ selection

*Patients *A total of 154 patients who underwent rectal resection through TaTME were reviewed for inclusion in the study. Fifty-seven patients were excluded: 20 underwent non-restorative procedures, 13 had not had stoma reversal yet, and 24 had stoma reversal surgery less than 6 months before the study period (Fig. 1).

Overall, 97 patients who underwent TaTME with primary anastomosis were included. All the demographic, clinical, operative, and postoperative features are outlined in Table 1.

**Table 1:** Clinical and pathological features of patients undergoing TaTME

	*n*	%
*Sex*		
Male	61	62.9
Female	36	37.1
Total	97	100.0
*Age (years)*		
Mean, SD	66.1	11.1
Median	68.0	
Range	36.0	86.0
*BMI*		
Mean, SD	25.3	3.8
Median	24.7	
Range	18.1	41.3
*ASA Score*		
ASA 1	9	9.3
ASA 2	79	81.4
ASA 3	9	9.3
Total	97	100.0
Distance from ARJ* (mm)		
Mean, SD	59.5	23.6
Median	60.0	
Range	15.0	120.0
*Neoadjuvant therapy*		
Yes	65.0	67.0
No	32.0	33.0
Total	97.0	100.0
*Diverting stoma*		
No	11.0	11.3
Ileostomy	82.0	84.5
Colostomy	4.0	4.1
Total	97.0	100.0
*Operative time (min)*		
Mean, SD	287.9	66.3
Median	280.0	
Range	180.0	573.0
*Stage*		
Stage 0	22.0	22.7
Stage 1	30.0	30.9
Stage 2	19.0	19.6
Stage 3	19.0	19.6
Stage 4	4.0	4.1
Other**	3.0	3.1
Total	97.0	100.0
*Anastomosis*		
Manual	4.0	4.1
Stapled end–end	81.0	83.5
Stapled side–end	12.0	12.4
Total	97.0	100.0
*Clavien–Dindo complications grades*		
C0	75.0	77.3
C1	11.0	11.3
C2	5.0	5.2
C3	6.0	6.2
C4	0.0	0.0
C5	0.0	0.0
Total	97.0	100.0
*Length of hospital stay (days)*		
Mean, SD	5.7	3.3
Median	5.0	
Range	3.0	23.0
*30-Day readmission*		
Yes	88.0	90.7
No	9.0	9.3
Total	97.0	100.0
Interval to stoma reversal (months)***		
Mean, SD	7.5	5.2
Median	6.3	
Range	0.5	31.1
Follow-up QoL evaluations (months)****		
Mean, SD	19.0	9.3
Median	18.1	
Range	6.6	46.2

*Anorectal junction (ARJ) measured on MRI

**Other = two patients with ulcerative colitis and one patient with Gardner Syndrome all requiring proctocolectomy

***Calculated on 86 patients with diverting stoma

****Interval between surgery and follow-up questionnaires aiming to evaluate quality of life (QoL) through assessing anorectal, urinary, and sexual functions

The mean age at the surgical procedure was 66.1 ± 11.1 years. Sixty-five patients (42 males and 23 females) received neoadjuvant chemoradiotherapy, and 32 patients were treated with upfront surgery. Ninety-three patients underwent resection with stapled colorectal anastomosis, while four patients underwent rectal intersphincteric resection with a handsewn colo-anal anastomosis; two patients underwent proctocolectomy with TaTME approach with ileal pouch–anal anastomosis (one for ulcerative colitis and one for Gardner Syndrome) and were included in the study as they were willing to participate in the functional outcomes analysis.

Eleven patients (11.3%) underwent surgery with primary anastomosis without diverting stoma. Four patients received a protective loop colostomy, while the anastomoses in the remaining 82 patients were protected using loop ileostomies. Only in one case was the transabdominal phase conducted by open surgery due to combined resection of liver metastasis. The mean operative time was 287.9 ± 66.3 min. Six percent of patients developed a Clavien–Dindo grade 3 complication with an anastomotic leak in 4.1%.

Among the 86 patients who received a protective stoma, the mean interval between surgery and stoma reversal was 7.5 ± 5.2 months. Overall, the postoperative follow-up for functional questionnaires was conducted at a median of 18.1 months (range 6.6–46.2 months).

*Anorectal functional outcomes* The analysis of the LARS questionnaire showed a mean total LARS score of 17.4 ± 13.8. In 97 patients, the percentages of those experiencing no LARS, minor LARS, and major LARS postoperatively were 60.8%, 13.4%, and 25.8%, respectively (Supplementary Table 3).

Statistical analyses revealed that age, operative time, and time to stoma reversal were correlated with LARS outcomes. Conversely, sex and neoadjuvant chemoradiotherapy were not correlated with LARS features (Table 2). However, mean LARS scores were documented to improve in females and in patients who underwent upfront surgery (mean LARS score 16.3 ± 14.1 versus 19.1 ± 13.3, and 14.3 ± 14.4 versus 18.9 ± 13.3, respectively; Supplementary Table 4) but no association was documented when correlating the previous ostomy presence with the occurrence of LARS symptoms (*P* = 0.3) (Supplementary Table 5).

**Table 2:** Univariable analyses for LARS and Wexner scores

LARS score	Median age, range	
No LARS	68.0 (59.5–74.5)	< 0.01*
Minor LARS	68.0 (62.0–69.0)	
Major LARS	68.0 (62.0–75.0)	

LARS score	Male *n* (%)	Female *n* (%)	
No LARS	38 (62.3%)	21 (58.3%)	0.7^§^
Minor LARS	7 (11.5%)	6 (16.7%)	
Major LARS	16 (26.2%)	9 (25.0%)	
Total	61 (100.0%)	336 (100.0%)	

LARS score	Neoadjuvant therapy *n* (%)	No neoadjuvant therapy *n* (%)	
No LARS	38 (58.5%)	21 (65.7%)	0.20^§^
Minor LARS	9 (13.8%)	4 (12.5%)	
Major LARS	18 (27.7%)	7 (21.8%)	
Total	65 (100.0%)	32 (100.0%)	

LARS score	Median operative time (min), IQR1–IQR3	
No LARS	280.0 (242.0–320.0)	< 0.01*
Minor LARS	268.0 (240.0–290.0)	
Major LARS	280.0 (254.0–318.0)	

LARS score	Median interval to stoma reversal (months), IQR1–IQR3	
No LARS	6.4 (4.0–10.0)	< 0.01*
Minor LARS	9.3 (3.2–12.5)	
Major LARS	5.1 (3.5–8.0)	

Wexner score	Median age (years), IQR1–IQR3	*P* value
No FI	63.0 (56.0–70.0)	< 0.01*
Minor FI	68.0 (60.0–76.0)	
Average and complete FI	70.0 (64.5–74.0)	

Wexner score	Male *n* (%)	Female *n* (%)	
No FI	22 (36.0%)	11 (30.5%)	0.67^§^
Minor FI	31 (50.8%)	18 (50.0%)	
Average and complete FI	8 (13.2%)	7 (19.5%)	
Total	61 (100.0%)	36 (100.0%)	

Wexner score	Neoadjuvant therapy *n* (%)	No neoadjuvant therapy *n* (%)	
No FI	17 (26.1%)	16 (50.0%)	0.01^§^
Minor FI	33 (50.8%)	16 (50.0%)	
Average and complete FI	15 (23.1%)	0 (0.0%)	
Total	65 (100.0%)	32 (100.0%)	

Wexner score	Median operative time (minutes), IQR1–IQR3	
No FI	285.0 (255.0–318.0)	< 0.01*
Minor FI	268.0 (240.0–318.0)	
Average and complete FI	280.0 (252.0–333.0)	

Wexner score (FT)	Median interval to stoma reversal (months), IQR1–IQR3	
No FI	6.2 (3.7–8.8)	< 0.01*
Minor FI	6.1 (3.5–10.5)	
Average and complete FI	7.4 (4.9–11.2)	

*Mann–Whitney *U* test

^§^Pearson’s chi-squared test

To evaluate if the learning curve could have an impact on LARS categories, the functional outcomes reported in the first TaTME procedures were compared with those obtained in the subsequent ones. Statistical analyses were conducted to balance the limited number of patients (post hoc analysis: effect size 0.3, alpha error 0.05, and power 1–beta 0.80). As documented in Table 3, when comparing the standardized residuals, there was no statistically significant difference between LARS categories in the first cohort of 27 patients and the following 70 patients.

**Table 3:** Learning curve and LARS scores

Categories		Patients 1–27	Patients 28–97
No LARS	Residual	0.73	−0.73
No LARS	*P* values	1	1
Minor LARS	Residual	2.24	− 2.24
Minor LARS	*P* values	0.14	0.14
Major LARS	Residual	−2.56	2.56
Major LARS	*P* values	0.06	0.06

The mean Wexner score among the 97 interviewed patients was 3.8 ± 4.8. Overall, 33 subjects (34.0%) reported no symptoms of FI, 49 patients (50.5%) fell in the category of minor FI, 10 patients (10.3%) were in the average FI group, while 5 (5.2%) showed a clinical picture of complete FI (Supplementary Table 3).

Statistical analyses confirmed that age, neoadjuvant chemoradiotherapy, operative time, and time to stoma reversal correlated with worse Wexner categories (Table 2 and Supplementary Fig. 1).

Although mean Wexner scores were similar in the male and female groups, a difference was documented in patients treated with upfront surgery compared with those treated with neoadjuvant chemoradiotherapy (mean Wexner score 1.8 ± 2.6 versus 4.7 ± 5) (Supplementary Table 4).

**Fig. 2:**
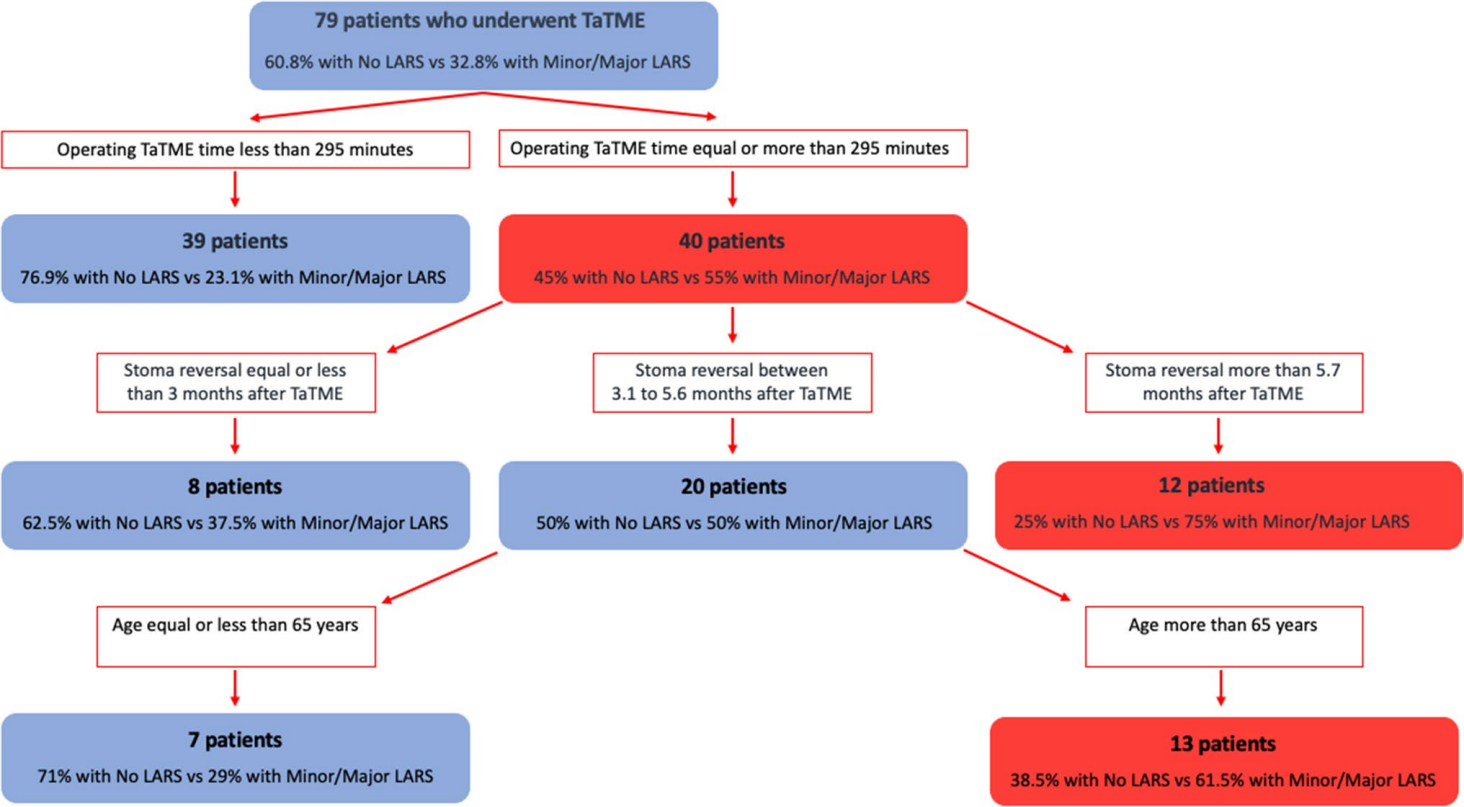
Random forest analysis

*Random forest analysis* This analysis focused on LARS questionnaires. The LARS score Random forest analysis showed an accuracy of 55–65%, whereas sensitivity and specificity were 50–65%. There was a prevalence of patients with LARS symptoms among the subjects who underwent a procedure longer than 295 min (55% no LARS versus 45% minor/major LARS). Within this subgroup, 75% of patients who underwent stoma reversal surgery after more than 5.6 months reported bowel impairment. Finally, in patients who had a procedure longer than 295 min, but who carried stoma for a time ranging from 3 to 5.6 months, those older than 65 years reported anorectal postoperative dysfunction (61.5% of patients). The algorithm with the functional outcomes based on these clinical features is presented in Fig. 2.

*Urinary and sexual functional outcomes* Univariate analysis showed a statistically significant correlation of urinary postoperative dysfunction and neoadjuvant chemoradiotherapy, both in the male and in the female population (Table 4). No correlation was documented between urinary function scores and age (Supplementary Fig. 2).

**Table 4:** Urinary functional outcomes

	Median ICIQ-MLUTS	IQR1–IQR3	*P* value	Median ICIQ-FLUTS	IQR1–IQR3	*P* value
*Neoadjuvant therapy*			< 0.01*			< 0.01*
Yes	4.0	(2.0–6.0)		4.0	(0.0–23.0)	
No	4.0	(2.0–18.0)		8.0	(2.7–14.0)	

*Mann–Whitney *U* test

Table 5 and Supplementary Figs. 3–4 report sexual functional outcomes. Worse median values were reported in the population treated with neoadjuvant chemoradiotherapy compared upfront surgery, both in males and in females. In particular, the male population reported poor outcomes, with the exception of erectile function, even though female patients reported higher percentage of sexual inactivity before surgery (56% versus 46%). Finally, elderly patients presented with worse sexual outcomes.

**Table 5:** Sexual functional outcomes

	Overall	Neoadjuvant therapy	No neoadjuvant therapy	*P* value
	Median, IQR1–IQR3	Median, IQR1–IQR3	Median, IQR1–IQR3	
*Male population*				
Erectile function	18.5 (1.0–30.0)	10.0 (1.0–28.0)	24.0 (2.5–28.5)	< 0.01*
Orgasm	6.0 (1.0–10.0)	6.0 (1.0–10.0)	8.0 (1.5–10.0)	< 0.01*
Sexual desire	6.0 (2.0–10.0)	5.0 (2.0–8.0)	8.0 (2.5–8.5)	< 0.01*
Satisfaction (intercourse)	5.5 (0.0–11.0)	0.0 (0.0–10.0)	10.0 (0.0–11.0)	0.047*
Satisfaction (overall)	6.0 (2.0–8.0)	3.0 (2.0–8.0)	8.0 (2.0–8.0)	< 0.01*
*Female population*				
FSFI	3.2 (2.0–17.5)	3.2 (2.0–4.7)	17.8 (2.0–25.0)	< 0.01*

*Mann–Whitney *U* test

## Discussion

This study documented favorable results in terms of anorectal functional outcomes after TaTME. When interviewed after surgery, the mean LARS score was lower than values from existing literature [7] and the vast majority of our patients reported minor fecal incontinence according to the Wexner score.

Aside from the recognised impact of neoadjuvant chemoradiotherapy [15], age, operative time, and time to stoma reversal significantly correlated with the incidence of bowel dysfunction. Since it was not possible to define a clear trend for all the categories, we computed this information to create an algorithm that could be useful to classify categories of patients undergoing TaTME.

 We explored the impact of the learning curve on the functional results: the cut-off values were based on a previous study from our group, which identified 27 as the number of TaTME required to significantly decrease the anastomotic leak rate [23]. This threshold was considered appropriate since leakage is a well-known factor impairing postoperative bowel function [24], and thus it was adopted in the present analysis. Interestingly, the TaTME learning curve did not have an impact on postoperative bowel function, although this comparison was limited by small numbers.

With respect to the secondary outcomes, postoperative urinary function significantly correlated to neoadjuvant therapy, independent of age. The same applies to sexual outcomes, even though, as expected, an increasing age was a relevant risk factor.

According to the literature, 19–52% of patients who underwent sphincter preserving rectal surgery for cancer experience altered defecation or LARS syndrome [7, 8, 14, 16, 25, 26]. The results of the present series revealed that a significant proportion of cohort had good function, with 13.4% of patients with minor LARS scores and 25.8% of patients with major LARS scores after TaTME, supporting the benefits of the transanal approach.

Undoubtedly, the transanal approach has been shown to provide a better visualization of the key zones where branches of the pelvic nerveplexus are located [27]. It therefore allows preservation of the autonomic innervation of the internal anal sphincter, the main area responsible for passive fecal continence [16]. However, the positive anorectal outcomes reported in our series could be explained by the strict selection of patients treated with restorative resections, indicated by the low number of colo-anal anastomoses, intersphincteric resections, and ileal pouch-anal anatomoses included in the cohort.

The height of the anastomosis, and consequently the length of remaining rectum, correlates with the risk of major LARS [11–14], since a significant loss of rectal volume can lead to an increased frequency and urgency to defecate [28].  Thickening of the rectal wall due to neoadjuvant radiation damage [28] can result in nerve impairment and similar poor function [29].

Despite many concerns related to the use of the transanal platform, with potential for anal stretching, prolonged dilatation, and risk of sphincter damage [8, 16, 30], different studies [7, 8, 20, 26, 31] have reported that laparoscopic TME and TaTME offer similar result in terms of functional outcomes.

A multicenter observational study [32] reported that the robotic approach may be superior in preserving postoperative anorectal function when compared with TaTME.  However,  this result may be influenced by the difference in the proportion of patients who underwent neoadjuvant chmeoradiotherapy among the groups analyzed.

Consistent with this evidence, patients from our series who underwent neoadjuvant therapy reported higher mean LARS and Wexner scores than those treated with upfront surgery.

Using a machine learning approach, we developed a statistical model to classify patients at risk of postoperative bowel impairment on the basis of clinical and operative data.

This random forest, combined with the existing preoperative risk scores such as the POLARS [33], may represent a valid clinical tool to offer proper preoperative counseling. It could be particularly useful in high-risk subsets of patients and may also guide a tailored therapeutic program (i.e., TAI-transanal water irrigation, biofeedback, electrostimulation, pelvic floor muscle training, and Kegel exercises [34]) in cases of delayed stoma reversal surgery. Evidence from the literature [35] reports that prompt application of these adjuncts (< 18 months from surgery) results in a greater improvement in fecal incontinence.

This study has some limitations: firstly, it is a single-center experience, and secondly patients were assessed only once after primary surgery or stoma reversal, without a baseline evaluation [36]. However, our group has a strong and consistent experience in TaTME, as documented by several publications in the field [22, 23, 37], and all the patients had a similar follow-up after surgery, so they can be regarded as homogeneous for long-term results. Also, the algorithm proposed here will require an external validation.

In conclusion, when performed in a high-volume center, TaTME can provide good long-term results for anorectal functions. Subgroups of patients with high-risk clinical features are at risk of developing major LARS syndrome; however, an algorithm with specific risk categories was developed and could be useful in the decision-making process, especially with respect to the timing of stoma reversal.

## Supplementary Information

Below is the link to the electronic supplementary material.

Supplementary file1 (DOCX 17445 KB)

## Data Availability

The data that support the findings of this study are available on request from the corresponding author. The data are not publicy available due to the privacy of research partecipants.
