# Supplementary material for: Functional outcomes after transanal total mesorectal excision (TaTME): a random forest analysis to predict patients’ outcomes
Source: Tech Coloproctol. 2023 Mar 5;27(11):1037–46. doi: 10.1007/s10151-023-02775-5 (PMC9985820; doi:10.1007/s10151-023-02775-5)
Supplement: Supplementary file 1 — Supplementary file1 (DOCX 17445 KB) [file 10151_2023_2775_MOESM1_ESM.docx]

**Functional outcomes after transanal total mesorectal excision (TaTME): a Random Forest analysis to predict patients' outcomes**

Flavio Tirelli MD, Laura Lorenzon MD PhD, Alberto Biondi MD*, Ilaria Neri MD, Gloria Santoro PhD, Roberto Persiani MD

^a^General Surgery Unit, Fondazione Policlinico Universitario Agostino Gemelli IRCCS, Rome, Italy

**Running Head.** Functional outcomes in TaTME surgery

**Corresponding author**. Alberto Biondi MD, General Surgery Unit, Fondazione Policlinico Universitario “Agostino Gemelli”, Catholic University, Largo Francesco Vito 1, 00168 Rome, Italy, email alberto.biondi@policlinicogemelli.it; phone number: +39-0630154974; fax +39-0630158015

**Supplementary Materials - Index**

| **Type - Supplementary Tables** |  |
| --- | --- |
| Supplementary Table 1 – STROBE checklist | *pag.2* |
| Supplementary Table 2 - Questionnaires | *pag.3* |
| Supplementary Table 3 **-** Anorectal functional outcomes | *pag. 5* |
| Supplementary Table 4 - LARS and Wexner scores according to sex and pre-operative treatments | *pag. 5* |
| Supplementary Table 5 **-** Association test between the variable “LARS” and The variable “ostomy presence” | *pag.5* |
| **Type - Supplementary Figures** |  |
| Supplementary Figure 1 - Postoperative anorectal functions in TaTME patients according to age | *pag. 7* |
| Supplementary Figure 2 - Postoperative urinary functional outcomes in TaTME patients according to age | *pag. 8* |
| Supplementary Figure 3- Post-operative Sexual functional outcomes in TaTME patients according to age (male population) | *pag. 9* |
| Supplementary Figure 4- Post-operative Sexual functional outcomes in TaTME patients according to age (female population) | *pag. 10* |
|  |  |

**Supplementary Table 1. STROBE checklist**


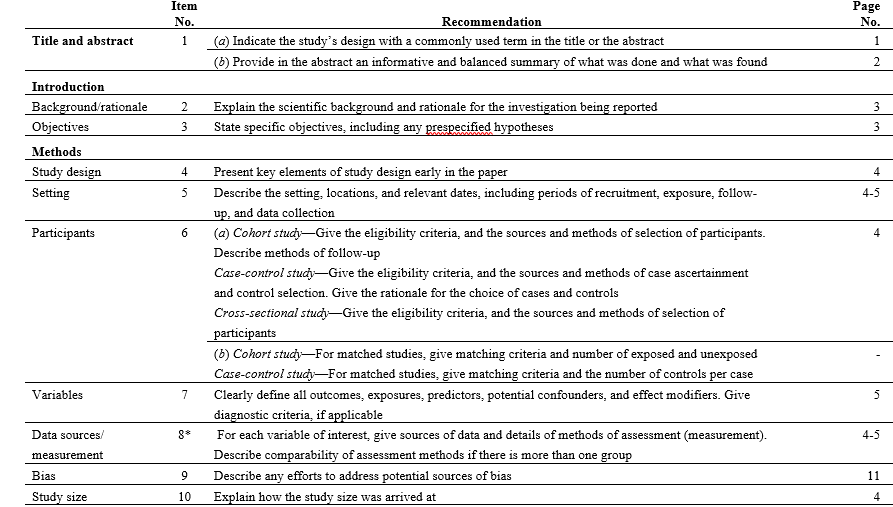


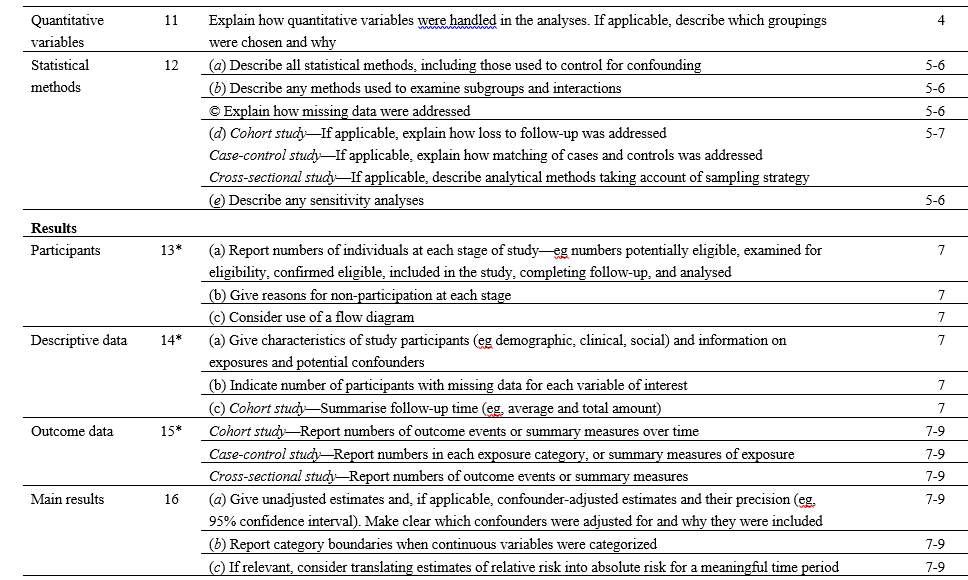


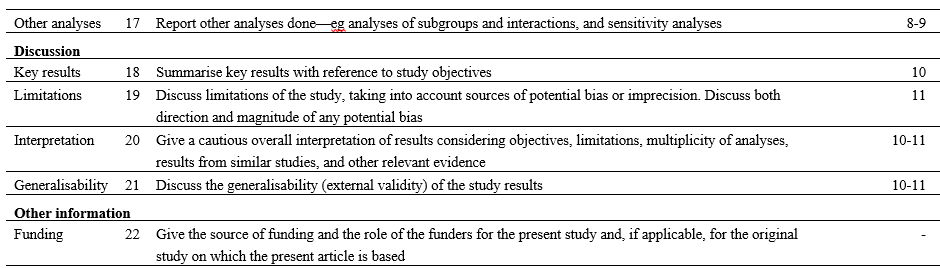


*Give information separately for cases and controls in case-control studies and, if applicable, for exposed and unexposed groups in cohort and cross-sectional studies.

**Note:** An Explanation and Elaboration article discusses each checklist item and gives methodological background and published examples of transparent reporting. The STROBE checklist is best used in conjunction with this article (freely available on the Web sites of PLoS Medicine at http://www.plosmedicine.org/, Annals of Internal Medicine at http://www.annals.org/, and Epidemiology at http://www.epidem.com/). Information on the STROBE Initiative is available at www.strobe-statement.org.

**Supplementary Table 2. Questionnaires**

| Questionnaire |  |
| --- | --- |
| CCFIS | Briefly, the CCFIS, also known as Jorge-Wexner scale, was designed to evaluate faecal incontinence. It is a five-items scoring system that investigates: solid incontinence, liquid incontinence, gas incontinence, pad wearing and lifestyle alterations. Every answer matches to a score ranging from 0 to 4, where 0 means “never” and 4 means “always”. The Wexner final score correspond to four categories: no faecal incontinence ("no FI", corresponding to Wexner score of 0), minor faecal incontinence ("minor FI" corresponding to Wexner score of 1-8); average faecal incontinence ("average FI" corresponding to Wexner score of 9-14), complete faecal incontinence ("complete FI" corresponding to Wexner score of 15-20). |
| LARS score | The LARS score is a symptom-based scoring system including five questions. It provides a general evaluation of rectal and bowel function after rectal surgery. Incontinence for flatus or liquid stools, high frequency of bowel motions, stool clustering and urgency are all symptoms of low anterior resection syndrome. The scores correspond to 3 LARS categories: no LARS (score range: 0-20), minor LARS (score range: 21-29) and major LARS (score range: 30-42). |
| ICIQ-MLUTS and ICIQ-FLUTS | ICIQ-MLUTS and ICIQ-FLUTS are questionnaires that evaluate the presence of the male lower urinary tract symptoms and their impact on quality of life (QoL), respectively in males and females. They are composed by 23 items, evaluating storage and emptying symptoms such as: frequency and urgency, presence of bladder pain or dysuria, nycturia, urinary incontinence (stress incontinence or unexplained incontinence) and pad usage, hesitancy and straining to start or to continue urination, urination position, strength of stream and weak stream history, intermittency of the stream, incomplete emptying and terminal dribbling, urinary retention and need of catheterization. Every answer matches to a score ranging from 0 to 4, where 0 means “never” and 4 means “all of the time”. For each item the questionnaires include a QoL question that consist in a Visual Analogue Scale (VAS). The VAS score doesn’t contribute to the overall score but it is intended as a clinical indicator of the overall bother the patient experiences. Overall score (ranging from 1 to 84) is obtained with greater values indicating increased symptoms severity. |
| IEF and FSFI | Male sexual function was assessed by the IIEF. It is a validated, multidimensional questionnaire composed by 5 items that investigate: erectile function, orgasm function, sexual desire, intercourse satisfaction and overall satisfaction. Questions refer to the 4 weeks leading up to the interview and answers consist in a 5-point Likert scale for each question. Erectile function in patients with sexual inactivity is not analyzed.  As far as women is concerned, FSFI is the most widely used scale for evaluating female sexual dysfunction and it was adopted for this analysis. It consists in 19 items that investigate: desire, excitation, lubrication, orgasm, satisfaction and dispareunia. Each answer matches to a score ranging from 0 or 1 to 5 points. The lowest final score is 2 and the highest is 36. A high score indicates a better sexual function. According to the authors, functional condition is considered good for FSFI score >30, moderate for FSFI score 23-29, poor for FSFI score <23. A score <26.55 is considered a Female Sexual Disorder (FSD). |

**Supplementary Table 3. Anorectal functional outcomes**

| LARS Score | n | % |
| --- | --- | --- |
| no LARS | 59.0 | 60.8 |
| minor LARS | 13.0 | 13.4 |
| major LARS | 25.0 | 25.8 |
| Total | 97.0 | 100.0 |
| WEXNER Score | **n** | **%** |
| no FI | 33.0 | 34.0 |
| minor FI | 49.0 | 50.5 |
| average FI | 10.0 | 10.3 |
| complete FI | 5.0 | 5.2 |
| Total | 97.0 | 100.0 |

**Supplementary Table 4. LARS and Wexner scores according to sex and pre-operative treatments**

|  | N patients | Mean Wexner Score; SD | Mean LARS Score; SD |
| --- | --- | --- | --- |
| Sex |  |  |  |
| Male | 61 | 3.4; 4.3 | 16.3; 14.1 |
| Female | 36 | 4.4; 5.5 | 19.1; 13.3 |
| Overall | 97 | 3.8; 4.8 | 17.4; 13.8 |
| Neoadjuvant Therapy |  |  |  |
| Yes | 65 | 4.7; 5.3 | 18.9; 13.3 |
| No | 32 | 1.8; 2.6 | 14.3; 14.4 |
| Overall | 97 | 3.8; 4.8 | 17.4; 13.8 |
|  |  |  |  |

**Supplemetary Table 5. Association test between the variable “LARS” and the variable “ostomy presence”**

| **Categories** |  | **Patients WITHOUT OSTOMY** | **Patients WITH**  **OSTOMY** |
| --- | --- | --- | --- |
| **No LARS** | **Residuals** | **-0.07** | **0.07** |
| **No LARS** | **p-values** | **1.0** | **1.0** |
| **Minor LARS** | **Residuals** | **-1.2** | **1.2** |
| **Minor LARS** | **p-values** | **1.0** | **1.0** |
| **Major LARS** | **Residuals** | **1.0** | **-1.0** |
| **Major LARS** | **p-values** | **1.0** | **1.0** |

**Supplementary Figures**

**
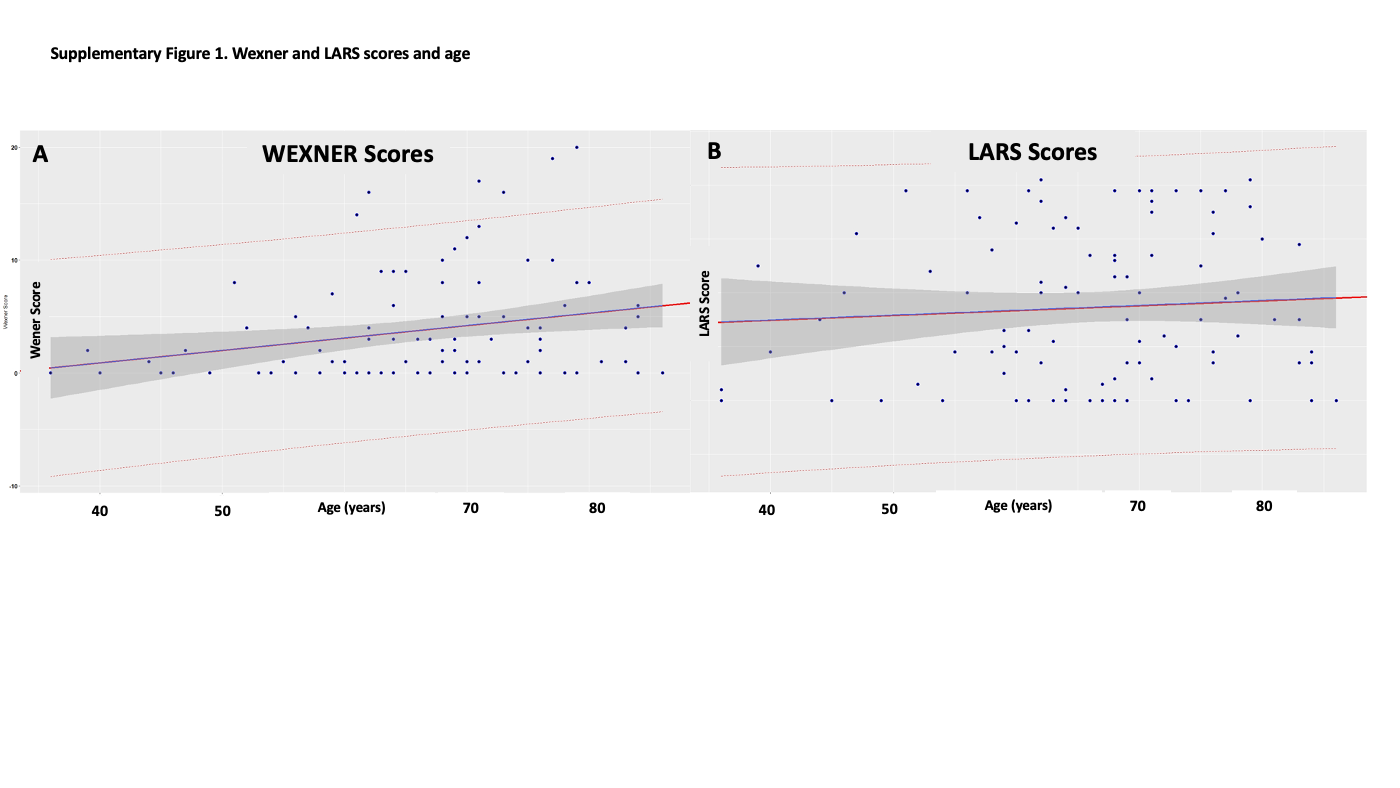
**

**Supplementary Figure 1.** Postoperative anorectal functions in patients who underwent TaTME: blue dots represent patients answers according to age; Red/Blue line represents intercept highlighted by the grey area and dashed red lines represent 95%CI. **A.** Wexner scores and patients' age**; B.** LARS scores and patients' age**.**

**
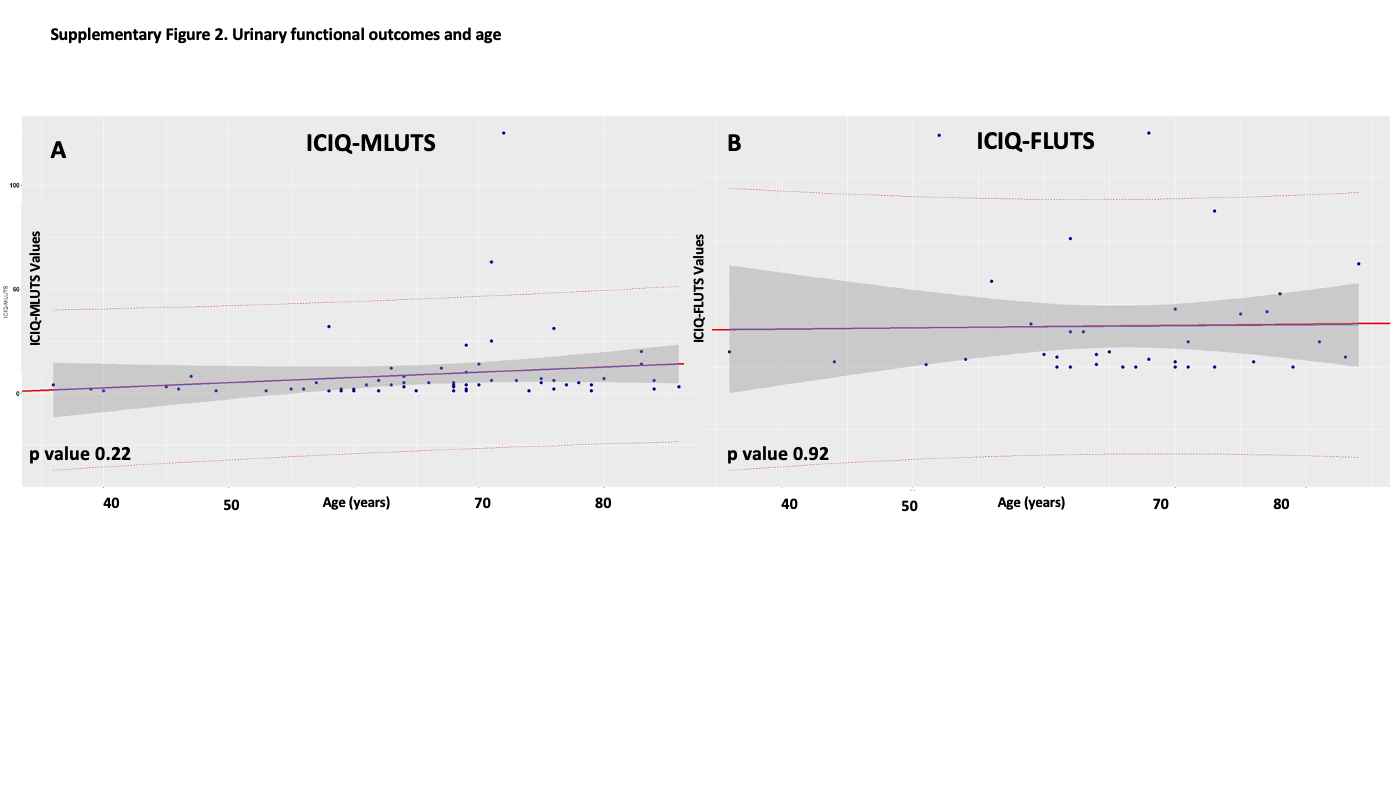
**

**Supplementary Figure 2.** Urinary functional outcomes and age in male population who underwent TaTME**.** Blue dots represent patients answers according to age; Red/Blue line represents intercept highlighted by the grey area and dashed red lines represent 95%CI; p values are reported for T Test results. **A.** ICIQ-MLUTS values in the male population and patients' age**; B.** ICIQ-FLUTS values in the female population and patients' age**.**

**
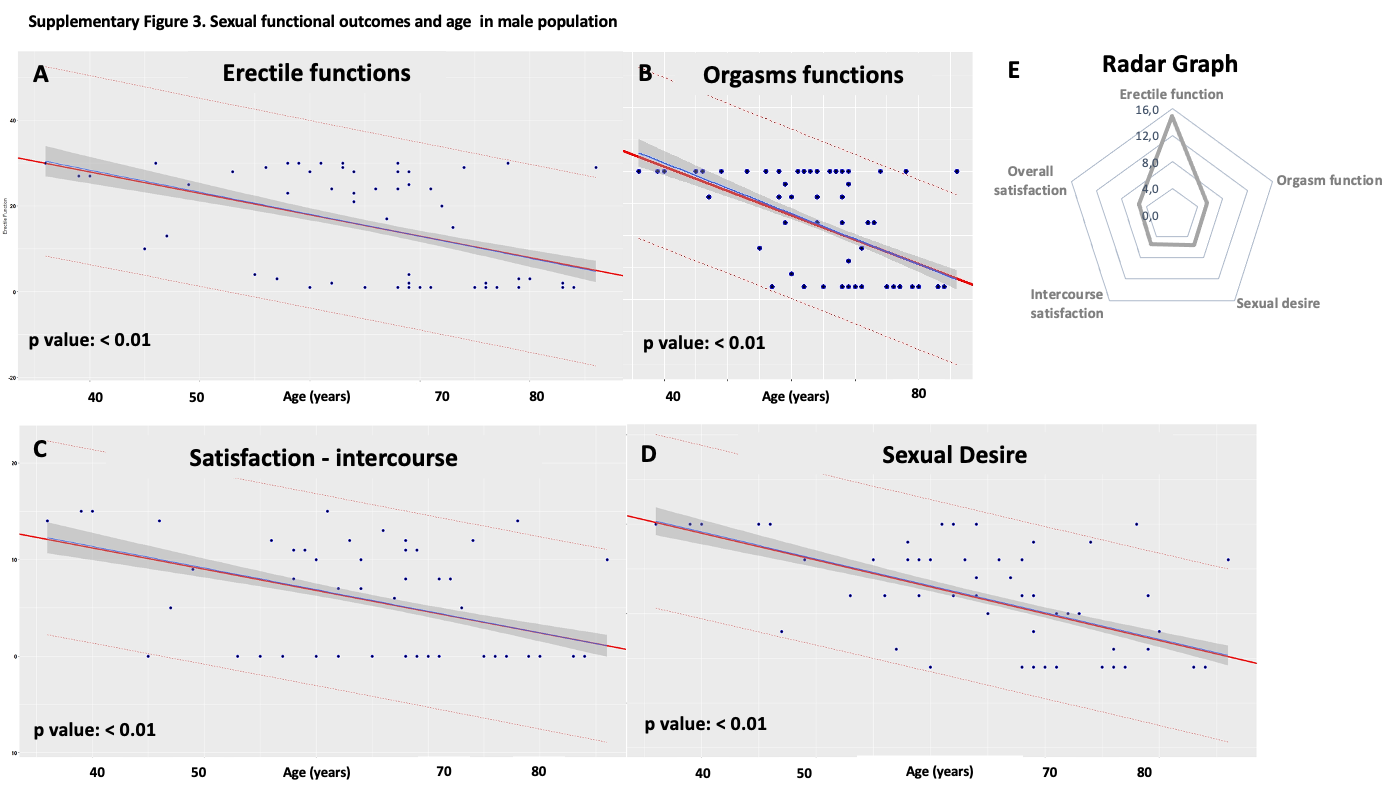
**

**Supplementary Figure 3.** Sexual functional outcomes and age in male population who underwent TaTME**.** Blue dots represent patients answers according to age; Red/Blue line represents intercept highlighted by the grey area and dashed red lines represent 95%CI; p values are reported for T Test results. **A.** Erection function and patients' age**; B.** Orgasms function and patients' age**. C.** Satisfaction of intercourse and patients' age**; D.** Sexual desire and patients' age**; E.** Radar graph reporting mean values for each item of the male sexual functions.

**
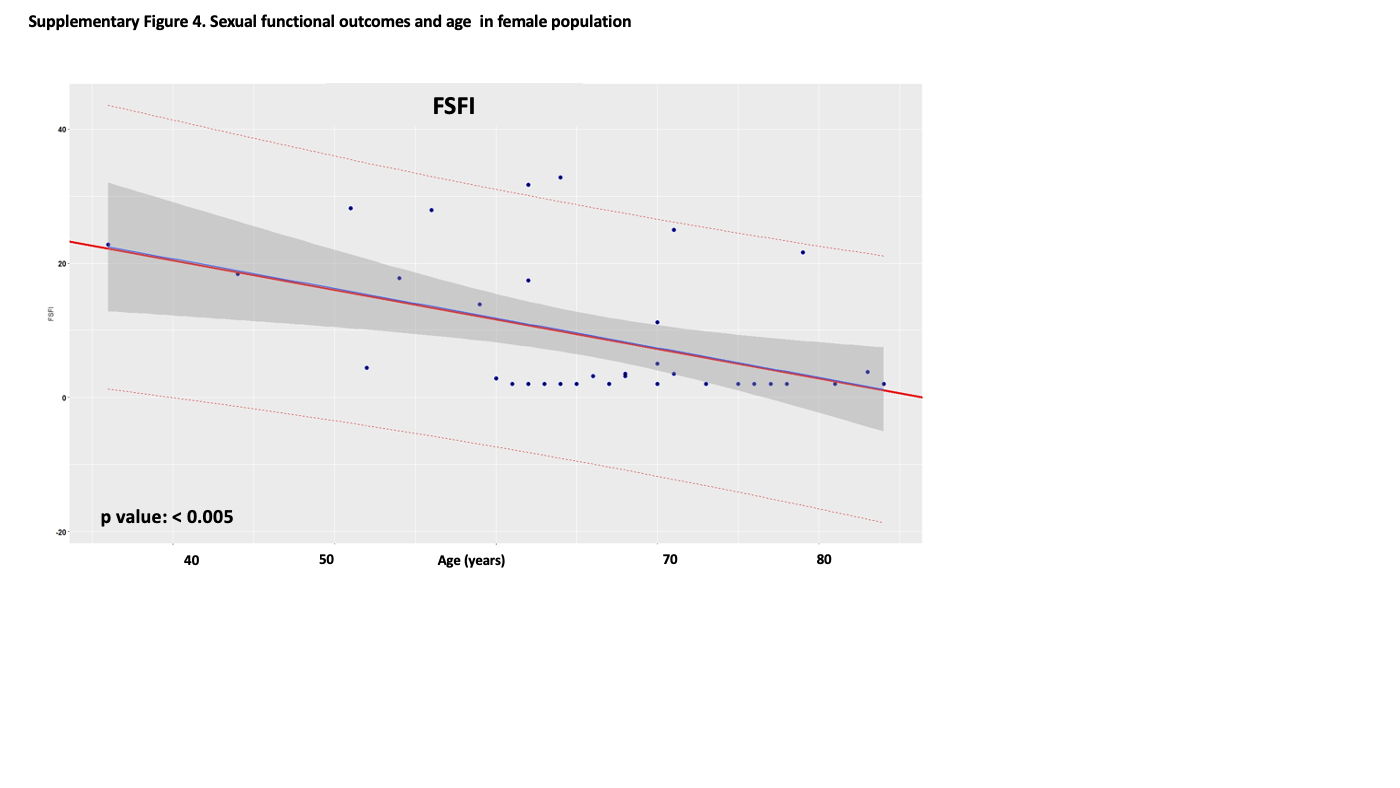
**

**Supplementary Figure 4.** Sexual functional outcomes and age in the female population who underwent TaTME**.** Blue dots represent patients answers according to age; Red/Blue line represents intercept highlighted by the grey area and dashed red lines represent 95%CI; p values are reported for T Test results.
